# A Specific Mixture of Fructo-Oligosaccharides and *Bifidobacterium breve* M-16V Facilitates Partial Non-Responsiveness to Whey Protein in Mice Orally Exposed to β-Lactoglobulin-Derived Peptides

**DOI:** 10.3389/fimmu.2016.00673

**Published:** 2017-01-12

**Authors:** Atanaska I. Kostadinova, Laura A. P. M. Meulenbroek, Betty C. A. M. van Esch, Gerard A. Hofman, Johan Garssen, Linette E. M. Willemsen, Léon M. J. Knippels

**Affiliations:** ^1^Division of Pharmacology, Utrecht Institute for Pharmaceutical Sciences, Utrecht University, Utrecht, Netherlands; ^2^Immunology, Nutricia Research, Utrecht, Netherlands

**Keywords:** acute allergic skin response, dietary interventions, food allergy, mouse models, oral tolerance, peptides, prevention, T cells

## Abstract

Oral tolerance is a promising approach for allergy prevention in early life, but it strongly depends on allergen exposure and proper immune environment. Small tolerance-inducing peptides and dietary immunomodulatory components may comprise an attractive method for allergy prevention in at-risk infants. This study aimed to investigate whether early oral exposure to β-lactoglobulin-derived peptides (BLG-peptides) and a specific synbiotic mixture of short- and long- chain fructo-oligosaccharides (scFOS/lcFOS, FF) and *Bifidobacterium breve* (*Bb*) M-16V (FF/*Bb*) can prevent cow’s milk allergy (CMA). Three-week-old female C3H/HeOuJ mice were orally exposed to phosphate buffered saline (PBS), whey protein, or a mixture of four synthetic BLG-peptides combined with a FF/*Bb*-enriched diet prior to intragastric sensitization with whey protein and cholera toxin. To assess the acute allergic skin response and clinical signs of allergy, mice were challenged intradermally with whole whey protein. Serum immunoglobulins were analyzed after a whey protein oral challenge. Cytokine production by allergen-reactivated splenocytes was measured and changes in T cells subsets in the spleen, mesenteric lymph nodes, and intestinal lamina propria were investigated. Pre-exposing mice to a low dosage of BLG-peptides and a FF/*Bb-*enriched diet prior to whey protein sensitization resulted in a significant reduction of the acute allergic skin response to whey compared to PBS-pretreated mice fed a control diet. Serum immunoglobulins were not affected, but anaphylactic symptom scores remained low and splenocytes were non-responsive in whey-induced cytokine production. In addition, preservation of the T_h_1/T_h_2 balance in the small intestine lamina propria was a hallmark of the mechanism underlying the protective effect of the BLG-peptides–FF/*Bb* intervention. Prior exposure to BLG-peptides and a FF/*Bb*-enriched diet is a promising approach for protecting the intestinal T_h_1/T_h_2 balance and reducing the allergic response to whole whey protein. Therefore, it might have implications for developing successful nutritional strategies for CMA prevention.

## Introduction

Food allergies are becoming a serious health concern worldwide. Cow’s milk allergy (CMA) is the most prevalent food allergy in infancy and the earliest to occur. Even though the majority of cow’s milk allergic infants outgrow their allergy (1), they are often affected by allergies to other foods, such as peanut; a phenomenon referred to as the “Food Allergic March” (2). CMA further plays an important role in the atopic march, meaning that early life CMA may predispose to inhalant-triggered hypersensitivity or asthma later in life (3).

Food allergy is suggested to occur due to defective oral tolerance (4). Oral tolerance is the process that actively suppresses the immune response to ingested harmless antigens (5, 6). Research is, therefore, focused on actively inducing or repairing oral tolerance. Studies in mice document that oral tolerance is a strongly antigen-dependent process (7), emphasizing the importance of antigen exposure in early life. Recent reports reveal that consumption of peanuts by Jewish infants in Israel is associated with fewer occurrences of peanut allergy (8) and that feeding at-risk infants the allergenic food early in life can prevent food allergy development (9). However, exposure to the intact antigen might also result in sensitization or allergic symptoms development in infants at high risk of developing food allergy. To avoid this, oral tolerance approaches implementing allergen fragments can be used instead. For instance, partially hydrolyzed whey proteins were shown to suppress allergic symptoms to cow’s milk in mice (10). Even small immunogenic peptides containing T cell epitopes can be potent inducers of tolerance and may be a suitable alternative to whole protein antigens (11). Supplementing hydrolyzed formula with specific peptides was found effective in preventing the allergic response to the native protein (12). In follow-up studies, a reduced allergic sensitization to the major cow’s milk protein β-lactoglobulin (BLG) was observed when it was co-administered with BLG-derived peptides (13). Meulenbroek et al. further reported that oral pre-exposure to synthetic BLG-derived peptides reduces the allergic skin response to whole whey protein (14). Altogether, these studies support the hypothesis that specific protein fragments may be suitable for allergen-specific immunomodulation.

Generic immunomodulation suggests the use of dietary components with beneficial immunomodulatory properties to support immune system maturation and natural oral tolerance development by providing the right environment for oral tolerance induction (15). Supplementing infant formulas with dietary prebiotics reduces the prevalence of atopic manifestations and induces bifidobacteria- and lactobacilli-predominating gut microbiota, resembling the situation in breast-fed infants (16–19). Bacterial dysbiosis, especially abnormal levels of bifidobacteria, has been associated with increased risk of allergy (20, 21). Interestingly, non-digestible oligosaccharides, such as inulin and other fructo-oligosaccharides, are selectively utilized by bifidobacteria in both rats and humans (22) and increase gut bifidobacteria by 10-fold in healthy volunteers (23). Studies in rodents suggest the involvement of T helper 1 (T_h_1) and T regulatory (T_reg_) cells in the prevention of allergic asthma and cow’s milk allergic symptoms by dietary non-digestible oligosaccharides (24, 25). Combining fructo-oligosaccharides with a probiotic bacterial strain such as *Bifidobacterium breve* (*Bb*) M-16V (a concept known as synbiotics) might result in stronger immunomodulatory properties. The M-16V bacterial strain is known for its anti-allergic activity (26, 27) and has shown beneficial effects in infants with atopic dermatitis (28). Further, the use of synbiotic mixtures containing *Bb* M-16V was found effective in preventing CMA allergic symptoms (29, 30) as well as house dust mite-induced allergic asthma in mice (31). In a recent clinical trial with infant formulas, it was reported that children fed a formula containing fructo-oligosaccharides and *Bb* M-16V had an increased percentage bifidobacteria in their feces (32). *Bb* M-16V is suggested to enhance the homing process of naïve T cells to mesenteric lymph nodes (MLN), induce mucosal IgA production (33), and upregulate the TGF-β1 signaling (34), while they can partially modulate the TNF-α signaling in epithelial cells (35). Therefore, a dietary synbiotic mixture combining fructo-oligosaccharides and *Bb* M-16V is of interest for providing generic immunomodulation in preventive strategies for food allergy.

In this study, we assessed the potential of an early oral exposure to a mixture of four synthetic BLG-derived peptides and a specific mixture of short- and long-chain fructo-oligosaccharides (scFOS/lcFOS, FF) and *Bb* M-16V (FF/*Bb*) to prevent allergy development in a murine model of orally induced CMA. We hypothesize that providing the right immune environment by means of a synbiotic diet during BLG-peptide presentation to immune cells would improve the capacity of the peptides to prevent allergic symptoms to the whole whey protein.

## Materials and Methods

### Peptides

**Figure 1:**
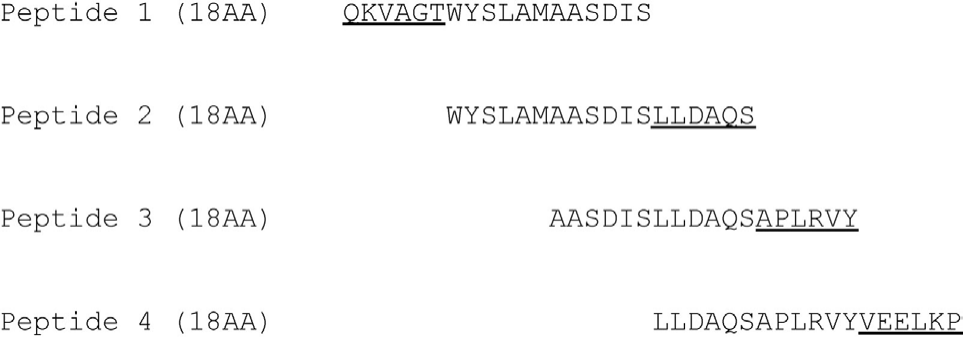
**Sequence information of the four synthetic peptides**.

Four 18-AA-long peptides from the B variant of BLG were synthetically produced by JPT Peptide Technologies (Berlin, Germany). The four peptides contain a 12-AA-long overlap (Figure 1) and were previously screened in human T cell lines and used in an animal model (14). The BLG-derived peptides were dissolved in sterile phosphate buffered saline (PBS; Lonza, Walkerville, MD, USA) and mixed until each peptide was at a concentration of 0.08 mg/mL (further referred to as PepMix).

### Diets

Semi-purified cow’s milk protein-free standard mouse chow was composed according to a AIN-93G recipe (control diet) and supplemented with 1% (wt:wt) of non-digestible scFOS (Raftilose P95, Beneo Orafti S.A., Oreye, Belgium) and lcFOS (Raftiline HP, Beneo Orafti S.A.) in a ratio 9:1 and 2% (wt:wt) 2 × 10^9^ CFU/g *Bb* M-16V (Morinaga Milk Industry, Tokyo, Japan) (FF/*Bb* diet; Research Diet Services, Wijk bij Duurstede, The Netherlands). The synbiotic components were mixed through the diet and pressed into pellets. Diets were stored at 4°C prior to use.

### Animals

Three-week-old pathogen-free female C3H/HeOuJ mice were purchased from Charles River Laboratories (Sulzfeld, Germany). Mice were housed in the animal facility of Utrecht University. This study was carried out in accordance with the recommendations of the Animal Ethics Committee of Utrecht University. The protocol was approved by the Animal Ethics Committee of Utrecht University (approval number DEC2014.II.12.100).

### Oral Tolerance Induction, Sensitization, and Challenge of Mice

**Figure 2:**
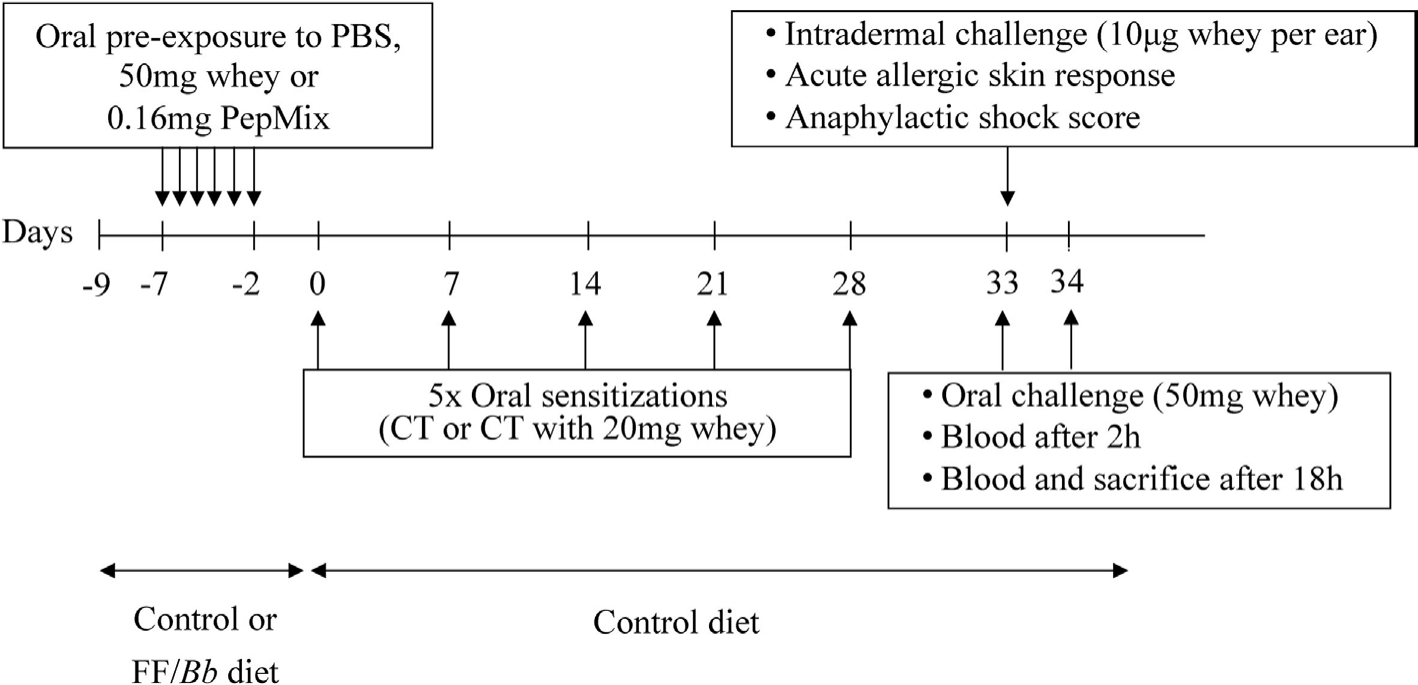
**A schematic overview of the murine model for cow’s milk allergy prevention**. CT, cholera toxin.

Upon arrival, mice were fed the control or the FF/*Bb* diet *ad libitum* for a period of 9 days (Figure 2). In the same week, mice were orally exposed (using a blunt needle) to 0.16 mg PepMix (0.04 mg of each BLG-derived peptide), 50 mg whey protein (DMV International, Veghel, The Netherlands), or PBS (0.5 mL) daily for 6 days. From day 0, all mice were maintained on control diet and sensitized as previously described (10). Five days after the last sensitization, an intradermal challenge was performed to assess the allergic response. Mice were challenged orally with 50 mg whey, followed by blood sampling at 2 h and blood sampling and sacrifice at 18 h after oral challenge. Serum was obtained and stored at −80°C until measurement.

### Evaluation of the Allergic Response

To measure the acute allergic skin response, mice were challenged intradermally in the ear pinnae with 10 µg whey protein per ear. Ear thickness was recorded before and 1 h after the intradermal challenge using a digital micrometer (Mitutoyo, Veenendaal, The Netherlands) and the acute skin response was calculated as Δ = ear thickness at 1 h − basal ear thickness and is expressed as delta micrometer. The anaphylactic shock symptoms were scored according to a previously described table (29).

### Allergen-Specific Immunoglobulins in Serum

Serum whey- and BLG-specific immunoglobulins were quantified by means of an enzyme-linked immunosorbent assay (ELISA) as previously described (10), with few modifications. Briefly, high binding Costar 9018 plates (Corning Inc., New York, NY, USA) were coated with 20 µg/mL whey or BLG protein in carbonate–bicarbonate coating buffer (Sigma-Aldrich, Zwijdrecht, The Netherlands) overnight at 4°C. Plates were washed, blocked for 1 h with 0.5% bovine serum albumin (Sigma-Aldrich)/0.05% Tween-20 (Merck, Billerica, MA, USA) buffer, and serum samples were incubated for 2 h at room temperature. After washing, 1 µg/mL of biotin-labeled rat anti-mouse IgE, IgG1, or IgG2a detection antibody (BD Biosciences, San Jose, CA, USA) was incubated for 1.5 h. The plates were washed and incubated for 1 h with streptavidin-horse radish peroxidase (Sanquin, Amsterdam, The Netherlands), washed again and the reaction was developed with 3,3′,5,5′-tetramethylbenzidine (TMB, Thermo Fisher Scientific, Waltham, MA, USA). The reaction was stopped with 2 M H_2_SO_4_, and absorbance was measured at 450 nm on a Benchmark plate reader (Bio-Rad, Veenendaal, The Netherlands).

### Short-Chain Fatty Acid (SCFA) Concentrations in Cecum

Cecal content was collected and immediately frozen in liquid nitrogen. Samples were stored at −80°C until measurement. Concentration of acetic, propionic, and butyric acids were determined by means of gas chromatography as previously described (36), using 2-ethylbutyric acid as internal standard.

### Cell Isolation from Tissues

Lymphocytes were isolated from spleen, MLN, and small intestine lamina propria. Spleens and MLN were crushed through 70 µm cell strainers. Splenocyte suspension was incubated with lysis buffer (8.3 g NH_4_Cl, 1 g KHC_3_O, and 37.2 mg EDTA dissolved in 1 L demi water and filter sterilized) to remove red blood cells and then resuspended in RPMI 1640, 10% fetal bovine serum and penicillin (100 U/mL)/streptomycin (100 µg/mL). For the isolation of the lamina propria cells, the small intestine was removed, cleared of Peyer’s patches, washed in cold PBS, opened longitudinally, and minced in 0.5-cm fragments. Samples were washed in Hank’s Balanced Salt Solution (HBSS; Invitrogen, Life Technologies, Carlsbad, CA, USA) containing 15 mM HEPES (Gibco, Life Technologies, Carlsbad, CA, USA), pH = 7.2 followed by 4 × 15-min incubations with HBSS supplemented with 15 mM HEPES, 5 mM Na_2_-EDTA, 10% fetal bovine serum, and penicillin (100 U/mL)/streptomycin (100 µg/mL), pH = 7.2. The fragments were washed in RPMI 1640, 5% fetal bovine serum and penicillin (100 U/mL)/streptomycin (100 µg/mL) and incubated 2 × 45 min with an enzyme solution containing 0.25 mg/mL Collgenase type VIII (Sigma-Aldrich). To collect the lamina propria cells, fragments were vortexed for 10 s after each incubation and passed through a 70 µm cell strainer.

### Flow Cytometry of Immune Cells

Spleen, MLN, and small intestine lamina propria cells were resuspended in PBS/1% bovine serum albumin and incubated with anti-mouse CD16/CD32 (Mouse BD Fc Block; BD Pharmingen, San Jose, CA, USA) to block non-specific binding sites. For surface staining, cells were incubated with CD4-PerCp-Cy5.5, CD69-APC, CXCR3-PE, CD25-AlexaFluor488, CD69-PE-Cy7, CD11c-PerCp-Cy5.5, CD8α-PE, CD11b-PE-Cy7, CD103-APC, CD11b-PE, CD83-FITC, CD86-APC (eBiosciences, San Diego, CA, USA), T1ST2-FITC (MD Biosciences, St. Paul, MN, USA), or CX3CR1-AlexaFluor488 (R&D Systems, Oxon, UK). Viable cells were distinguished by means of a fixable viability dye eFluor^®^ 780 (eBioscience). For detecting transcription factors, cells were first fixed and permeabilized with Foxp3 Staining Buffer Set (eBioscience) according to manufacturer’s protocol and then stained with Foxp3-APC and RorγT-PE antibodies (eBioscience). Results were collected with BD FACSCanto II flow cytometer (Becton Dickinson, Franklin Lakes, NJ, USA) and analyzed with FlowLogic software (Inivai Technologies, Mentone, VIC, Australia).

### *Ex Vivo* Restimulation Assay and Cytokine Production

Spleens were removed aseptically and single cell suspensions were obtained as described above. Splenocytes (6 × 10^5^) were cultured either with medium or with 500 µg/mL whey protein for 5 days at 37°C, 5% CO_2_. Supernatants were collected and analyzed for IL-5, IL-13, IL-10, IL-17A, and IFN-γ by means of a Cytometric Bead Array Flex Set assay (BD Biosciences) following manufacturer’s instructions. Results were obtained with BD FACSCanto II flow cytometer and analyzed with FCAP v.3.0 software (Becton Dickinson).

### Statistical Analysis

For all statistical analyses, GraphPad Prism 6.0c software for Macintosh (GraphPad Software, San Diego, CA, USA) was used. Anaphylactic shock scores and serum immunoglobulins were analyzed by Kruskal–Wallis test followed by Dunn’s *post hoc* test for seven pre-selected comparisons. All other data were analyzed by one-way ANOVA, followed by Bonferroni’s multiple comparison *post hoc* test for selected groups. SCFA data and splenocytes cytokine results were first LOG-transformed. For testing correlations non-parametric Spearman correlation coefficient test was used. All data are presented as mean ± SEM of 4–8 animals per group. *p* < 0.05 was considered of statistical significance.

## Results

### Prior Exposure to BLG-Derived Peptides and FF/*Bb* Diet Reduced the Acute Allergic Skin Response to Whey Protein

**Figure 3:**
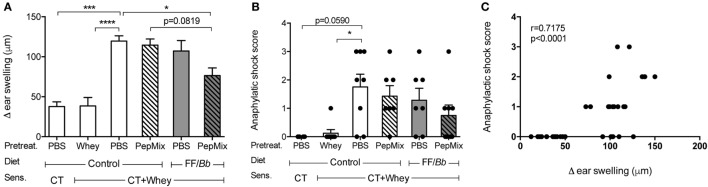
**Allergic response to whey protein after intradermal exposure**. Five days after the last sensitization, mice were challenged intradermally with whey and the acute allergic skin response **(A)** and anaphylactic shock **(B)** were determined. The two parameters were analyzed also in a correlation model **(C)**. Data are presented as mean ± SEM; *n* = 4 in PBS/CT group and *n* = 6–8 in all other groups; *p < 0.05, ***p < 0.001, ****p < 0.0001; **(A)** was analyzed with one-way ANOVA followed by Bonferroni *post hoc* test for selected groups; **(B)** was analyzed with Kruskal–Wallis non-parametric test, followed by Dunn’s *post hoc* test for selected groups, and **(C)** was analyzed with non-parametric Spearman correlation coefficient test. CT, cholera toxin.

To study the capacity of early exposure to dietary components and/or BLG-peptides to prevent CMA, mice were first pretreated for six consecutive days, then orally sensitized to whey protein with cholera toxin as an adjuvant and finally challenged intradermally with whey. Upon intradermal challenge with whey, PBS-pre-exposed whey-sensitized (PBS/CT+Whey) allergic controls developed a significantly higher acute allergic skin response compared to the sham-sensitized control mice (PBS/CT). However, administering PepMix while feeding mice the FF/*Bb* diet significantly reduced the acute skin response compared to the PBS/CT+Whey group fed the control diet (*p* < 0.05, Figure 3A). Prior exposure to the PepMix alone or feeding mice only the FF/*Bb* diet for 9 days was insufficient to prevent the allergic skin response. However, combining the FF/*Bb* diet with the PepMix pretreatment, tended to improve its preventive capacity when compared to mice exposed to PepMix and fed the control diet (*p* = 0.0819). As a control for maximal oral tolerance induction, animals were pre-exposed to a high dose of whey protein prior to sensitization which resulted in the strongest reduction of the acute allergic skin response as expected (Figure 3A). PBS/CT+Whey allergic control mice fed the control diet during the pretreatment period, tended to develop more severe anaphylactic shock symptoms after intradermal whey challenge compared to the sham-sensitized controls (*p* = 0.0590) (Figure 3B). Mice administered the PepMix while fed the FF/*Bb* diet still experienced allergic symptoms but to a lesser extent. In line with this, the suppressed acute allergic skin response was found to correlate with less severe anaphylactic shock symptoms (Figure 3C).

### Pretreatment with BLG-Peptides and FF/*Bb* Does Not Affect the Whey- or BLG-Specific Immunoglobulins

As type I food allergy is associated with increased levels of serum immunoglobulins (37), the effect of the pretreatments on whey- and BLG-specific immunoglobulin production was determined 18 h after the oral challenge with whey protein (Table 1). Even though all immunoglobulins were elevated in the PBS/CT+Whey allergic control mice, only whey-specific IgE levels were significantly elevated compared to the sham-sensitized control mice. BLG-specific IgE and IgG1 were elevated significantly compared to the whey-pretreated mice. Though not significantly reduced, the levels of BLG-IgE detected in the combined PepMix+FF/*Bb* group resembled the levels measured in the sham-sensitized controls.

**Table 1:** **Whey- and BLG-specific immunoglobulin levels in serum**.

Pretreatment Sensitization		PBS CT	Whey Whey + CT	PBS Whey + CT	PepMix Whey + CT	FF/*Bb* Whey + CT	PepMix + FF/*Bb* Whey + CT
Whey-specific	IgE (AU)	0.0** (0.0–0.3)	4.0** (0.0–16.7)	168.9 (85.7–180.8)	104.0 (58.4–203.1)	131.4 (41.0–189.3)	65.7 (0.0–96.8)
	IgG1 (AU × 1,000)	0.0 (0.0–0.0)	0.0 (0.0–0.0)	3,267.0 (976.6–4,063.0)	6,580.0 (2,822.0–7,459.0)	3,750.0 (86.2–5,253.0)	1,221.0 (0.0–5,592.0)
	IgG2a (AU × 1,000)	3.4 (0.0–10.7)	2.7 (0.0–41.8)	417.0 (288.0–595.2)	1,470.0 (282.8–3,341.0)	1,048.0 (30.6–1,412.0)	141.9 (0.0–745.3)
BLG-specific	IgE (AU)	4.5 (4.2–5.2)	3.6* (3.1–3.7)	88.0 (21.7–130.3)	48.1 (6.1–178.8)	20.7 (16.0–74.5)	5.2 (2.8–13.9)
	IgG1 (AU × 1,000)	26.5 (20.8–30.2)	20.5* (15.3–46.7)	2,386.0 (515.6–6,469.0)	6,303.0 (3,260.0–8,883.0)	2,654.0 (393.6–5,994.0)	1,959.0 (29.9–7,802.0)
	IgG2a (AU × 1,000)	4.3 (0.8–10.6)	0.0 (0.0–10.6)	357.1 (203.6–738.6)	1,488.0 (339.2–3,562.0)	294.3 (42.0–871.7)	102.4 (1.6–663.2)

Data represent median (25%–75% IQR). *p < 0.05, **p < 0.01 when compared to PBS-pretreated whey-sensitized allergic controls, analyzed with Kruskal–Wallis non-parametric test after removing outliers as indicated by Tukey.

*BLG, β-lactoglobulin; CT, Cholera toxin; FF/Bb, short- and long-chain fructo-oligosaccharides and Bifidobacterium breve M-16V*.

### FF/*Bb* Increased the SCFA Levels in Cecum

**Figure 4:**
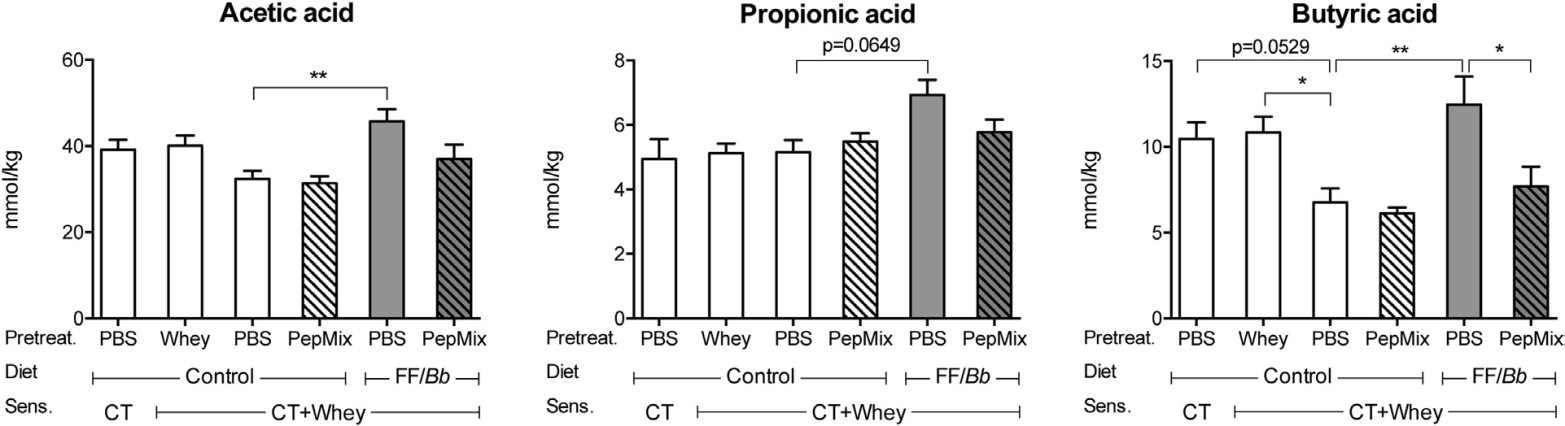
**Short-chain fatty acids (SCFA) concentrations in cecum**. On day 34, the cecum content was collected for measuring SCFA in supernatants. The absolute amount of acetic, propionic, and butyric acid is presented. Data are presented as mean ± SEM for *n* = 4 in PBS/CT group and *n* = 6–8 in all other groups. *p < 0.05, **p < 0.01 as analyzed with one-way ANOVA followed by Bonferroni *post hoc* test for selected groups after LOG transformation of the data. CT, cholera toxin.

To monitor for changes in the microbiota, concentrations of SCFA in the cecum were determined as a measure of microbiota metabolic activity. PBS/CT+Whey allergic controls tended to have reduced butyric acid concentrations compared to sham-sensitized control mice (*p* = 0.0529) and significant lower levels than the Whey-tolerized CT+Whey-sensitized controls. Feeding mice the FF/*Bb* diet for only 9 days prior to allergic sensitization resulted in increased SCFA concentrations in the gut after the 5-week sensitization period compared to the PBS/CT+Whey allergic controls, reflecting that the diet possibly stimulated the microbiome metabolic activity. Prior exposure to FF/*Bb* not only prevented the drop in butyric acid but also increased acetic acid and tended to increase propionic acid concentrations (Figure 4).

### Combined Exposure to PepMix and FF/*Bb* Prevented a T_h_1/T_h_2 Disbalance in the Intestinal Lamina Propria

**Figure 5:**
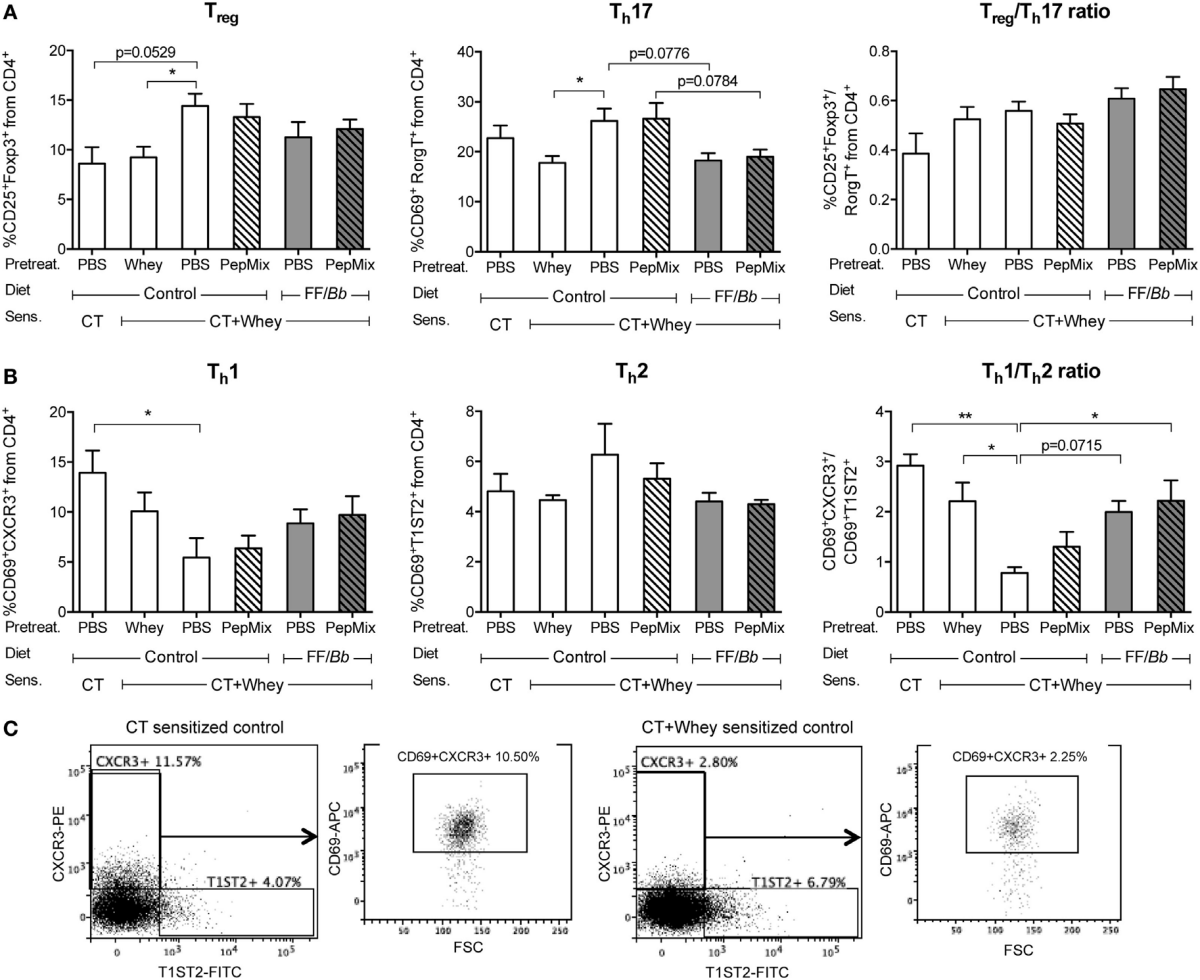
**T cell subsets in the small intestine lamina propria**. Lymphocytes from the small intestine lamina proria were isolated and analyzed by flow cytometry for T_reg_ and T_h_17 phenotype **(A)** or for T_h_1 and T_h_2 phenotype **(B)**. Graphs illustrate the T cell subtypes as percentage of the CD4^+^ population and the T_reg_/T_h_17 and T_h_1/T_h_2 ratios within the CD4^+^ population. **(C)** Representative FACS plots for the percentages total T_h_1 and T_h_2 cells from the CD4^+^ population in sham-sensitized and whey-allergic mice as well as the percentages activated T_h_1 (CD69^+^CXCR3^+^) cells from the CD4^+^ in the same mice. Data are presented as mean ± SEM of *n* = 4 in the PBS/CT group and *n* = 6–8 in all other groups. *p < 0.05, **p < 0.01 as analyzed with one-way ANOVA followed by Bonferroni *post hoc* test for selected groups; CT, cholera toxin.

In order to investigate the local effects in the intestine, the small intestine lamina propria lymphocytes were isolated and different T cell (Figure 5) and DC (Figure S1 in Supplementary Material) subsets were analyzed by flow cytometry. A tendency for higher percentage of T_reg_ cells was observed in the PBS/CT+Whey allergic mice fed the control diet compared to the sham-sensitized mice, while pretreatment with whole whey protein prevented this increase (Figure 5A). The frequency of activated T_h_17 cells did not differ between the sham-sensitized mice and the PBS/CT+Whey allergic controls; however, feeding whey protein prior to sensitization resulted in a reduced percentage of activated T_h_17 cells, and, similarly, pre-exposure to the FF/*Bb* diet with or without PepMix tended to decrease the T_h_17 frequency. Furthermore, PBS/CT+Whey allergic mice had a lower frequency of activated T_h_1 cells, while the percentage of activated T_h_2 cells remained high (Figure 5B). As a result, the activated T_h_1/T_h_2 cell balance was shifted in favor of T_h_2 in these allergic mice. By contrast, prior oral exposure to PepMix and FF/*Bb*, similar to pretreatment with whole whey protein, helped preserving the T_h_1/T_h_2 balance. Feeding mice only the FF/*Bb*-supplemented diet tended to preserve the balance (*p* = 0.0715). When investigating the DC population in the small intestine lamina propria, no changes in the frequency of CD11c^+^ DC cells expressing the CD83 activation marker or the CD86 co-stimulatory molecule were observed (Figure S1A in Supplementary Material). Furthermore, the percentage of T_h_1 polarizing CD11b^−^CD8^+^ lymphoid DC or T_reg_ and T_h_2 polarizing CD11b^+^CD8a^−^ myeloid DC (38–40) were not affected (Figure S1B in Supplementary Material). The higher percentage T_reg_ cells in the PBS/CT+Whey group fed the control diet did not coincide with enhanced frequency of the T_reg_-inducing CD103^+^ DC (39, 41) (Figure S1C in Supplementary Material).

### Cytokine Production in Spleen Cells upon Whey Stimulation Is Silenced by the Combined Exposure to PepMix and FF/*Bb*

**Figure 6:**
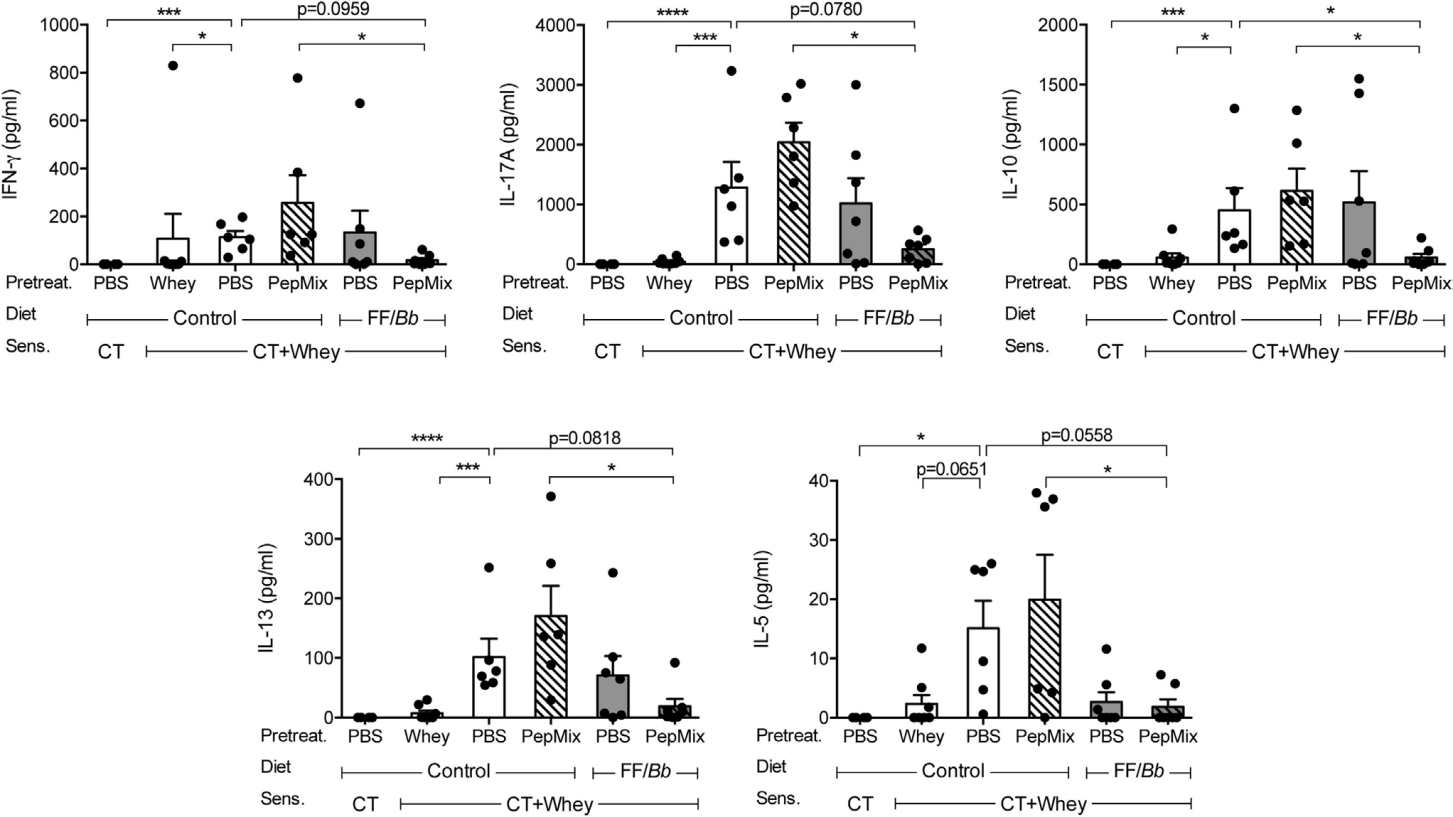
**Impact on T cell function in the spleen**. *Ex vivo* cytokine production by splenocytes after stimulation with allergen for 5 days. Data are presented as mean ± SEM of *n* = 4 in PBS/CT group and *n* = 6–8 in all other groups after subtracting the background cytokine levels. *p < 0.05, ***p < 0.001, ****p < 0.0001 as analyzed with one-way ANOVA followed by Bonferroni *post hoc* test for selected groups after LOG transformation of the data. CT, cholera toxin.

To investigate whether the preventive treatments affect lymphocyte phenotype, spleen and MLN cells were analyzed by flow cytometry. Furthermore, splenocytes were stimulated *ex vivo* with allergen for 5 days to determine their allergen-induced cytokine profile as a measure of effect on cell functionality in the systemic compartment. In contrast to the local effects in the small intestine, lymphocyte phenotypes in the spleen and the MLN were not affected (Figure S2 in Supplementary Material). However, splenocytes from PBS/CT+Whey allergic mice markedly increased the production of IL-13, IL-5, IL-10, IFN-γ, and IL-17A, while prior exposure to whole whey protein prevented this increase completely (Figure 6). Similarly, feeding mice a combination of PepMix and FF/*Bb* diet rendered splenocytes less responsive to *ex vivo* whole whey stimulation. Especially, it significantly reduced IL-10 (*p* < 0.05). Also whey-induced production of IL-5, IL-13, IL-17A, and IFN-γ remained low and also tended to be reduced when compared to the PBS/CT+Whey allergic controls (*p* < 0.10 for all). Importantly, when compared to the PepMix treatment alone, the combination of PepMix and FF/*Bb* diet resulted in significantly lower IFN-γ, IL-17A, IL-10, IL-13, and IL-5 secretion (Figure 6).

## Discussion

The present study demonstrates that a short pre-exposure to a low-dose mixture of four BLG-derived T cell epitopes was able to stimulate the development of oral tolerance to the major cow’s milk protein whey only when combined with a FF/*Bb* synbiotic diet. This oral tolerance was defined as the ability to reduce the acute allergic skin response after intradermal challenge with whole whey protein. Distinctive mechanistic features related to the preventive effect include the silencing of cytokine production upon *ex vivo* whole whey protein restimulation and the maintenance of the intestinal T_h_1/T_h_2 balance.

The concept of using BLG-derived peptides for oral tolerance has previously been explored in animal models (12–14). In addition, synbiotics have been shown to alleviate allergic symptoms. However, in contrast to previous reports, this study focuses on (1) a short period of dietary intervention prior to sensitization and (2) low dose of a mixture of four short BLG-derived peptides for inducing tolerance to the whole whey protein. In our study, mice were exposed to the synbiotic diet only for 9 days before and not during the sensitization, while Schouten et al. provided the synbiotic diet 2 weeks prior to and continued throughout the sensitization period (29). Furthermore, in this study, a mixture of four specific BLG sequences was used for oral tolerance induction, while other studies co-administered BLG-peptides combined with the whole BLG protein (13) or with BLG hydrolyzate (12). Only in one previous study, Meulenbroek et al. have documented a preventive effect of the BLG-derived peptides alone, but a 100-fold higher dose of the peptides was administered compared to the dose used in the current study (14). Here, we report that pre-exposure to a much lower dose of BLG-derived peptides was able to reduce the acute allergic skin response to the whole whey protein only when combined with a FF/*Bb* dietary intervention. Therefore, supplementing a BLG-peptide-based preventive treatment with dietary synbiotics helps to reduce the amount of peptides needed to prevent the allergic response after whole whey sensitization. In addition to the dose, other factors, such as the frequency of administration, tolerogenic potency, and T cell receptor affinity, should be considered for optimizing the magnitude of oral tolerance induction and allergic response inhibition (11, 42).

Feeding the FF/*Bb*-enriched diet promoted the efficacy of the BLG-peptides and, therefore, emphasizes the importance of the proper immune environment during antigen presentation and oral tolerance induction in early life. Beneficial protective effects of the FF/*Bb* synbiotics have previously been shown in a murine model of allergic asthma (31). In CMA, Schouten et al. showed that dietary intervention with a similar synbiotic mixture, containing galacto- and fructo-oligosaccharides and *Bb* M-16V (GF/*Bb*), before and during sensitization with whey protein, was effective in preventing the allergic response in mice by improving the T_h_1 response (29, 43). This is in accordance with our data where combining the PepMix with FF/*Bb* diet supported the local preservation of the T_h_1/T_h_2 balance in the intestine. Furthermore, previous reports have documented that feeding inulin and other fructo-oligosaccharides positively influences the colonization of the gut with bifidobacteria (23). Even when combined in a synbiotic mixture with *Bb* M-16V, an increase in fecal bifidobacteria percentage was reported in infants (32). It is not excluded that the FF/*Bb* has influenced the colonization with bifidobacteria during the pre-exposure with peptides and in such way has contributed to providing more tolerance-prone environment. However, future studies need to explore the microbiota composition during the tolerance induction period. Furthermore, the exposure to the *Bb* M-16V *via* the diet might have contributed to a more tolerogenic and anti-allergic environment by enhancing mucosal IgA production (33) and/or upregulating the TGF-β1 signaling in the gut (34). More in-depth studies are needed to confirm if these mechanisms are involved in the preventive effect of the combined PepMix-FF/*Bb* approach.

Dietary interventions with non-digestible oligosaccharides have been shown not only to affect the gut microbiota composition but also its metabolic activity (43). In the current study, mice were exposed to a FF/*Bb*-enriched diet for 9 days prior to sensitization and were fed a control diet during the 6-week sensitization period. After this period, increased levels of SCFA microbiota metabolites were measured in the cecum of mice fed the FF/*Bb* diet. However, butyrate levels were reduced in PBS/CT+Whey allergic mice fed the control diet and also in mice pretreated with PepMix and FF/*Bb*. Since PBS/CT+Whey allergic mice fed the control diet had significantly lower levels of butyrate compared to sham controls and whey-pretreated tolerant mice, this indicates that the allergic phenotype as such may reduce the butyrate levels in the mice. This may imply that the microbiome was changed or the metabolism of butyrate in the mucosal tissue was altered. The PepMix+FF/*Bb*-treated group, on the other hand, was successfully but only partially protected against the allergic sensitization which may relate to the lower butyrate levels observed in this group. This suggests the involvement of a preventive mechanism of PepMix+FF/*Bb* that is independent of the cecal butyric acid concentrations measured at the end of the experiment. Nevertheless, it is not excluded that the SCFA levels were differentially modulated during the PepMix+FF/*Bb* pretreatment period and this had contributed to the protective effect (44). However, further investigation needs to confirm this possibility. Most importantly, a short exposure to the FF/*Bb* diet alone was not sufficient to modulate the systemic allergen-specific response and to prevent the acute allergic response, emphasizing the importance of co-administering the peptides for oral tolerance induction to the antigen.

Pre-exposure to the combination of FF/*Bb* and BLG-peptides modulated the T cell responses to whole whey to a greater extend than the B cell responses. In a murine model of CMA, it was demonstrated that the whole whey protein was able to prevent both whey-specific immunoglobulins and allergic symptoms while the partially hydrolyzed protein was able to prevent the effector response only (10). Further, previous *in vivo* preventive studies with peptides (11, 12, 14) as well as therapeutic studies with peptide immunotherapy (42, 45), reported no effect on allergen-specific IgE levels in serum.

Main effector mechanisms of oral tolerance are active suppression and T cell anergy/deletion (46). Low antigen doses may favor active suppression *via* regulatory cells while high antigen doses may favor T cell anergy/deletion; however, these mechanisms are not mutually exclusive (47). In the current study, mice were exposed to a high dose of whey protein or to a low dose of PepMix combined with FF/*Bb* diet prior to sensitization. Strikingly, the results of those two treatments were very comparable on the level of intestinal T_h_-cell balance and on the *ex vivo* whey-induced cytokine production of splenocytes. Feeding mice whole whey protein or PepMix+FF/*Bb* resulted in preservation of the homeostatic T_h_1/T_h_2 balance rather than expansion of Foxp3^+^ T_reg_ cells. The local response in the intestine, however, suggested increased percentage of CD25^+^Foxp3^+^ T cells in the PBS/CT+Whey allergic mice that was reversed in the whey-pretreated tolerant mice. This effect was, however, not coincided by increase in the T_reg_-inducing CD103^+^ DC subtype. Overall, no clear modulating effect on the different DC populations could be demonstrated at the end of the experiment. However, the late-phase at which the DC phenotyping is performed might not reflect the situation during the early induction phase where mice were fed the FF/*Bb* diet and given the BLG-peptides. In line with this, Hacini-Rachinel et al. reported a transient increase of ICOS^+^CD25^+^Foxp3^+^ T cells after 5 days of oral pre-exposure to egg protein which was detectable only within the first days after the last oral pre-exposure (48). It is, therefore, of importance to further examine the effects on DC and T cell differentiation directly after the tolerance induction period.

Cytokines are important mediators in sensitization and establishment of tolerance (49). The whey protein pretreatment alike Perrier et al.’s was found to prevent the allergen-induced production of T_h_2-associated cytokines as well as IL-17A and IFN-γ in whey-sensitized mice (49). Combining the BLG-peptides treatment with the FF/*Bb* synbiotic diet silenced the allergen-induced cytokine production by splenocytes. This is in line with the cytokine suppression in peptide-induced tolerance described in previous studies (11, 50, 51). Importantly, the whey-induced cytokine production by splenocytes in the combined PepMix and FF/*Bb* group was significantly lower than in the group pretreated with BLG-peptides and fed the control diet. This underscores the essential contribution of the synbiotic diet in silencing of the allergen-specific T-cell response in mice exposed to PepMix. Furthermore, it highlights the importance of the right immune environment during oral tolerance induction with the low dose BLG-peptides which is crucial for the splenic non-responsiveness to whey re-exposure, potentially resulting from anergy. However, more in-depth investigation is necessary to confirm this mechanism for allergy prevention by BLG-peptides and FF/*Bb*.

In conclusion, this study demonstrated a reduction of the allergic response to the whole whey protein after a combined pre-exposure to only four synthetic BLG-derived peptides and a FF/*Bb*-enriched diet. The preventive effect of this combined pretreatment was associated with a preserved intestinal T_h_1/T_h_2 balance and whey-induced non-responsiveness of splenocytes. Therefore, specific peptides and FF/*Bb* synbiotics may have implications as dietary supplementation for early life food allergy prevention. Further studies are needed to confirm the potential of this approach to prevent food allergy in at-risk infants.

## Author Contributions

AK, LW, and LK conceptualized the study; BE, GH, and LM participated in the animal study; AK acquired and analyzed the data and drafted the manuscript; LK, LW, and JG revised the manuscript critically and provided overall supervision.

## Conflict of Interest Statement

None of the authors have a competing financial interest in relation to the presented work; LK and LM are employed at Nutricia Research; and BE and JG are partly employed at Nutricia Research. AK received funding from Nutricia Research.

## Abbreviations

BLG, β-lactoglobulin; CMA, cow’s milk allergy; CT, cholera toxin; ELISA, enzyme-linked immunosorbent assay; FF/*Bb*, specific synbiotic mixture of short- and long-chain fructo-oligosaccharides and *Bifidobacterium breve* M-16V; MLN, mesenteric lymph nodes; PBS, phosphate buffered saline; PepMix, mixture of four BLG-derived peptides; SCFA, short-chain fatty acids; Th, T helper cells; Treg, regulatory T cells.
