# Supplementary material for: A Specific Mixture of Fructo-Oligosaccharides and Bifidobacterium breve M-16V Facilitates Partial Non-Responsiveness to Whey Protein in Mice Orally Exposed to β-Lactoglobulin-Derived Peptides
Source: Front Immunol. 2017 Jan 12;7:673. doi: 10.3389/fimmu.2016.00673 (PMC5226939; doi:10.3389/fimmu.2016.00673)
Supplement: Supplementary file 1 [file Image_1.PDF]

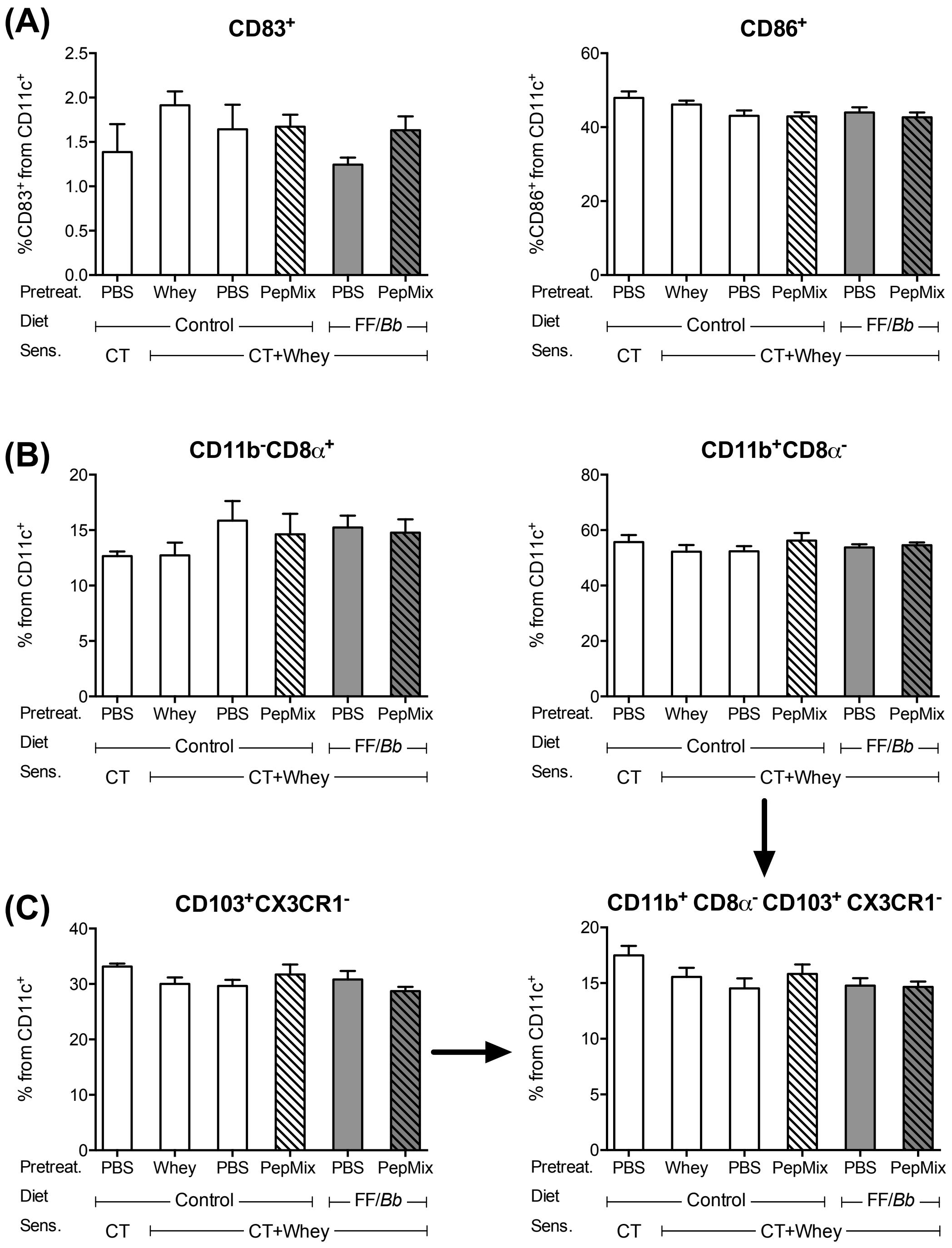


**Figure S1** Effect on DC subsets in small intestine lamina propria. Frequency $CD83^+$ and $CD86^+$ cells in the $CD11c^+$ population (A), percentages $T_h1$-polarizing $CD11b^-CD8\alpha^+$ lymphoid DC and $T_h2$- or $T_{reg}$–polarizing $CD11b^+CD8\alpha^-$ myeloid DC (B) and $T_{reg}$-inducing $CD103^+$ DC (C) populations from the $CD11c^+$ are graphed; Data are presented as mean ± SEM of n=4 in the PBS/CT group and n=6-8 in all other groups.
