# Supplementary material for: A Specific Mixture of Fructo-Oligosaccharides and Bifidobacterium breve M-16V Facilitates Partial Non-Responsiveness to Whey Protein in Mice Orally Exposed to β-Lactoglobulin-Derived Peptides
Source: Front Immunol. 2017 Jan 12;7:673. doi: 10.3389/fimmu.2016.00673 (PMC5226939; doi:10.3389/fimmu.2016.00673)
Supplement: Supplementary file 2 [file Image_2.PDF]

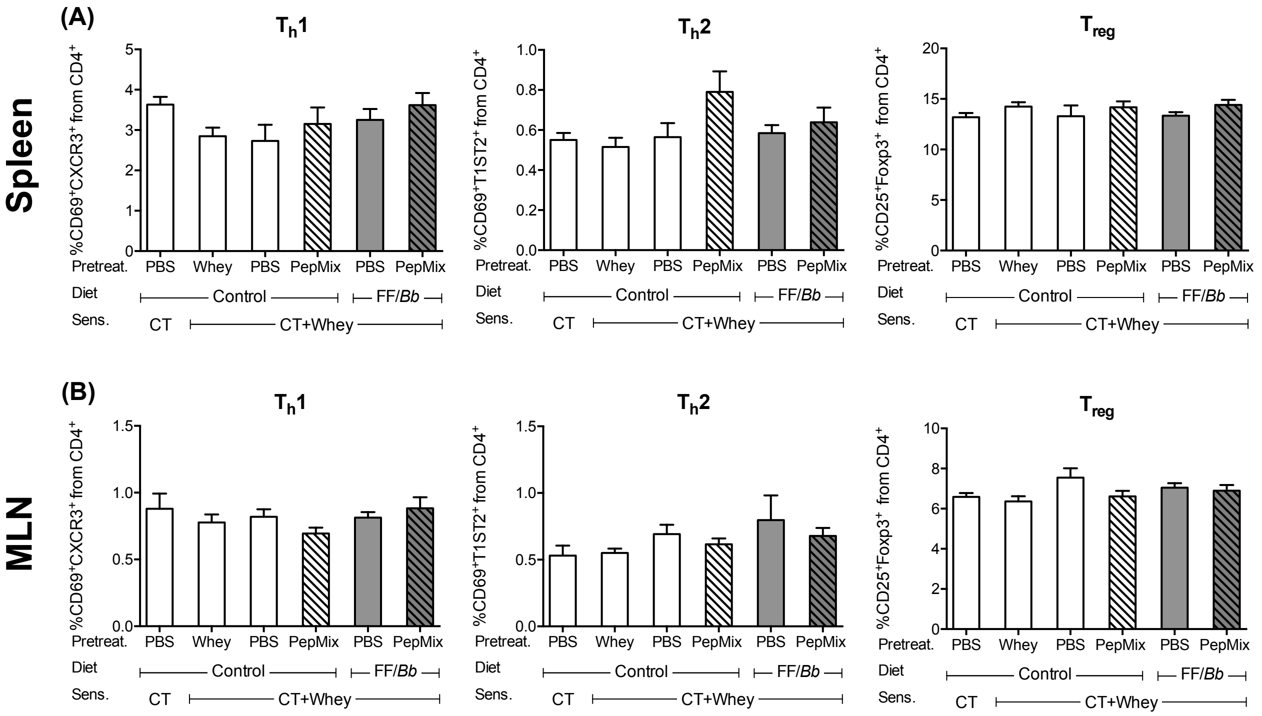

Figure S2 Effect on T cell subsets in spleen and MLN. Percentages T_h1, T_h2 and T_{reg} cells from the $CD4^+$ population in spleen (A) and MLN (B); Data are presented as mean ± SEM of n=4 in the PBS/CT group and n=6-8 in all other groups.
